# Side‐by‐side versus stent‐in‐stent technique for stent deployment during systemic chemotherapy in biliary tract cancer patients with malignant hilar biliary obstruction

**DOI:** 10.1002/deo2.70075

**Published:** 2025-02-07

**Authors:** Shunsuke Imamura, Kazuo Watanabe, Kanae Inoue, Tomonao Taira, Taro Shibuki, Tomoyuki Satake, Shota Yamaguchi, Mitsuhito Sasaki, Hiroshi Imaoka, Shuichi Mitsunaga, Masafumi Ikeda

**Affiliations:** ^1^ Department of Hepatobiliary and Pancreatic Oncology National Cancer Center Hospital East Chiba Japan

**Keywords:** biliary tract cancer, chemotherapy, malignant hilar biliary obstruction, side‐by‐side, stent‐in‐stent

## Abstract

**Objectives:**

With the improved prognosis of patients with biliary tract cancer (BTC) owing to advances in chemotherapy, long‐term stent patency has become an important goal in patients undergoing biliary stent placement. We compared the duration of stent patency between unresectable BTC patients undergoing multi‐stenting for malignant hilar biliary obstruction by the side‐by‐side (SBS) and stent‐in‐stent (SIS) techniques during systemic chemotherapy.

**Methods:**

We retrospectively evaluated the data of 62 unresectable BTC patients who underwent multi‐stenting before the first or second cycle of first‐line chemotherapy. Stent deployment was performed by the SBS technique in 40 patients (SBS group) and by the SIS technique in 22 patients (SIS group).

**Results:**

The median time‐to‐recurrent biliary obstruction was 147 days in the SBS group and 252 days in the SIS (*p* = 0.029), being longer in the SIS group. The rates of development of early adverse events were 28% and 9% (*p* = 0.09) and the rates of development of late adverse events were 26% and 14% in the SBS and SIS groups (*p* = 0.27). The median overall survival was 480 days in the SBS group and 563 days in the SIS group (*p* = 0.92).

**Conclusion:**

The duration of stent patency was shorter in the SBS group than in the SIS group; thus, the SIS technique is preferable to the SBS technique for biliary stent deployment in unresectable BTC patients during systemic chemotherapy.

## INTRODUCTION

Multiple or bilateral metallic stenting is widely used for liver drainage and palliation in malignant biliary obstruction (MBO).^1^, ^2^, ^3^, ^4^ The side‐by‐side (SBS) and stent‐in‐stent (SIS) techniques are the main methods used for multi‐stenting in patients with hilar MBO.^5^, ^6^ While the SBS technique is relatively easy for stent placement and re‐intervention, it is prone to bile duct overexpansion. The SIS technique allows more natural placement with less overexpansion, although breakage through the stent mesh could be a challenge.^7^ There are also reports of no significant difference in the duration of patency between SBS and SIS,^8^, ^9^ reports of longer patency in SBS,^10^ and reports of longer patency in SIS,^11^ all of which are inconclusive. Choosing between SBS and SIS for hilar MBO remains controversial due to varied patient backgrounds, including cancer types and chemotherapy regimens. Therefore, we compared SBS and SIS stent patency during chemotherapy for unresectable biliary tract cancer (BTC). Since a longer duration of uninterrupted chemotherapy can improve a patient's prognosis, we also examined the effect of stenting on the duration of chemotherapy and number of chemotherapy interruptions needed in the patients.

## METHODS

### Study Design

This was a single‐institutional retrospective study. The Institutional Review Board of the National Cancer Center (#2020‐209) approved the study, which adhered to the Declaration of Helsinki. The ethics committee of our hospital waived the need to obtain consent from the patients since this work involved retrospective use of anonymized patient data.

### Patients

Between January 2016 and August 2021, 896 unresectable BTC patients were admitted to our hospital. The patient inclusion criteria were as follows: (1) patients with histopathologically diagnosed BTC who were receiving systemic chemotherapy; (2) patients who underwent multi‐metallic stenting of the hilar bile duct; (3) patients in whom stenting was performed prior to induction or second cycle of first‐line chemotherapy. To assess the effect of stent patency and chemotherapy, we extracted patients who had undergone stent placement before the first or second cycle of first‐line chemotherapy. The exclusion criteria were as follows: (1) plastic stent placement; (2) no stent placement; (3) surgically altered anatomy; (4) distal bile duct stent placement; (5) unilateral stent placement; (6) no chemotherapy planned; and (7) stent inserted after the second cycle of first‐line chemotherapy. The SBS technique was mainly used in 2016–2019 and the SIS technique in 2020–2021.

### Procedures

**FIGURE 1:**
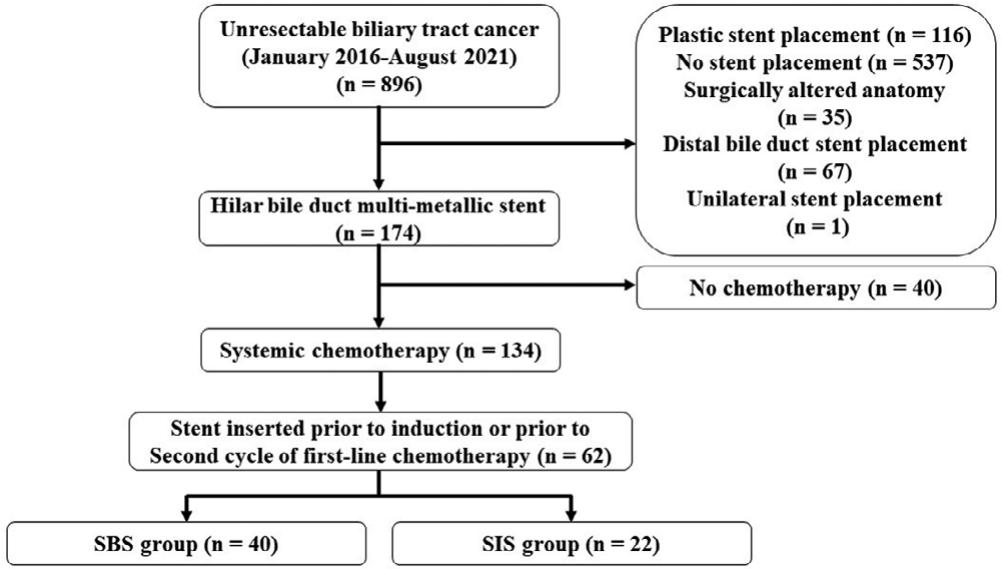
(a) In the side‐by‐side (SBS) deployment method, two self‐expanding metallic stents (SEMSs) were placed in parallel in the common bile duct after inserting two guidewires. (b) In the stent‐in‐stent (SIS) deployment method, the second guidewire was inserted through the mesh of the first SEMS, resulting in the overlapping of the two SEMSs in the common bile duct.

First, two guidewires were inserted into the bile ducts. In the SBS method for stent deployment, the first self‐expanding metallic stent (SEMS) was placed on one side over the guidewire, followed by the placement of the second SEMS on the other side. Both the SEMSs were oriented in parallel in the common bile duct (Figure 1a). For SIS deployment, after the first SEMS had been inserted into one side over the guidewire, insertion of the guidewire employed for placement of the first SEMS into the other side via the central open mesh of the first SEMS was performed using, as a landmark, the guidewire that had been inserted in the other side before insertion of the first SEMS. The two SEMS overlapped in the common bile duct (Figure 1b). Uncovered braided SEMSs with diameters of 8 or 10 mm and lengths sufficient to cover the stenotic area were selected. Endoscopic sphincterotomy was performed in all the patients who underwent stent placement. Re‐intervention typically involved placing an inner plastic stent after balloon extraction of ductal debris. Endoscopists with less than 5 years of experience in endoscopy were defined as trainees, and those with experience of 5 years or over were defined as experts.

### Chemotherapy

The chemotherapy regimens used for first‐line therapy varied and included gemcitabine alone (GEM), gemcitabine plus cisplatin (GC), GC plus S‐1 (GCS), and GC plus an investigational drug. All patients received at least one cycle of chemotherapy and none received external radiation therapy. Oncologists selected regimens based on patient condition and complication risk.

### Follow‐up and definitions of events

Data on recurrent biliary obstruction (RBO), clinical success, and adverse events (AEs) were defined according to the TOKYO criteria.^12^ Clinical success was defined as serum bilirubin level normalization or a ≥50% decrease within 14 days, or <75% of the pretreatment value within 1 month.^9^, ^10^ Early and late AEs were defined as any stent or procedure‐related AEs developing within and after 30 days of SEMS placement, respectively. RBO was defined as stent occlusion or migration and time‐to‐recurrent biliary obstruction (TRBO) as the length of time between the SEMS placement and development of RBO. Censoring in TRBO was defined as death without stent occlusion or termination of follow‐up. Stent occlusion was defined as recurrence of jaundice and cholestasis and/or evidence of biliary dilation on imaging studies, necessitating biliary re‐intervention. Time‐to‐treatment failure (TTF) was defined as the period from the start date of chemotherapy to the final administration date of first‐line chemotherapy. Overall survival (OS) is defined as the length of time from the start of treatment until death from any cause. Censoring in OS was defined as the end of follow‐up without observed death.

### Statistical analysis

Categorical variables were compared by the chi‐square or Fisher's exact test, and continuous variables by the Student *t*‐test or Wilcoxon rank‐sum test. A Cox proportional hazards model was used to estimate the hazard ratios and 95% confidence intervals (95% CIs). A *p*‐value <0.05 in a two‐sided test was considered as denoting statistical significance. TRBO was estimated using the Kaplan–Meier method and compared between groups using the log‐rank test. Deaths without RBO were treated as censored at the time of death. R software, version 4.1.3 was used for all the statistical analyses.

## RESULTS

**FIGURE 2:**
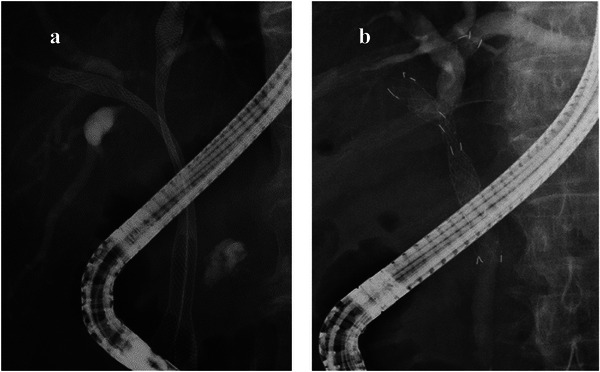
A flowchart showing patient selection. A review of our database identified 174 patients who underwent hilar multi‐stenting. We included 62 patients who had stent placement prior to the initiation or second cycle of first‐line chemotherapy. Among these 40 patients received stent placement using the side‐by‐side technique, while 22 patients received the stent‐in‐stent technique.

A review of our database revealed that a total of 174 patients had undergone hilar multi‐stenting. We extracted 62 patients who had undergone stent placement prior to the initiation or second cycle of first‐line chemotherapy; of these, the stent deployment was performed by the SBS technique in 40 patients, and by the SIS technique in 22 patients. The median follow‐up period was 287.5 days. A flowchart of patient recruitment for the present study is shown in Figure 2. The clinicopathological characteristics of the patients are shown in Table 1. While there were fewer patients with metastatic disease in the SBS group than in the SIS group (*p =* 0.018), there was no statistically significant difference in the sex, age, performance status, cancer type, Bismuth classification,^13^ chemotherapeutic regimens used, or the number of patients who had undergone endoscopic sphincterotomy between the two groups.

**TABLE 1:** Comparison of the clinical characteristics between the side‐by‐side and stent‐in‐stent groups.

	SBS	SIS	*p*‐value
Number of patients, *n* (%)	40 (65)	22 (35)	
Sex, *n* (%)			0.35
Male	30 (75)	14 (64)	
Female	10 (25)	8 (36)	
Age (years)			0.67
Median (range)	70.5 (50–88)	72.5 (34–86)	
Performance status, *n* (%)			0.66
0	39 (98)	21 (95)	
1/2	1 (3)	1 (5)	
Cancer type, *n* (%)			0.35
PHC	12 (30)	11 (50)	
DCC	6 (15)	1 (5)	
ICC	5 (13)	1 (5)	
GBC	17 (43)	9 (41)	
Stage, *n* (%)			0.018
Locally advanced	27 (68)	8 (36)	
Metastatic	13 (33)	14 (64)	
Bismuth type, *n* (%)			0.57
I	5 (13)	1 (5)	
II	29 (73)	18 (82)	
III	6 (15)	3 (14)	
Chemotherapeutic regimen, *n* (%)			0.16
GC	23 (58)	6 (27)	
GC+ ID	5 (13)	5 (23)	
GCS	11 (28)	10 (45)	
GEM	1 (3)	1 (5)	
Best response, *n* (%)			
CR	0 (0)	1 (5)	
PR	3 (8)	2 (9)	
SD	29 (73)	13 (59)	
PD	8 (20)	5 (23)	
NE	0 (0)	1 (5)	
Objective response rate (%)	7.5	13.6	0.66
Disease control rate (%)	80.0	72.7	1.00
Timing of chemotherapy initiation, *n* (%)			
Post stent placement	33 (83)	22 (100)	0.10
History of endoscopic sphincterotomy, *n* (%)			
Present	31 (78)	13 (59)	0.15
Follow‐up period (days)			
Median (range)	275 (59–1062)	296 (83–955)	0.64

Abbreviations: DCC, distal cholangiocarcinoma; ICC, intrahepatic cholangiocarcinoma; GBC, gallbladder carcinoma; GC, gemcitabine plus cisplatin; GCS, gemcitabine, cisplatin plus S‐1; GEM, gemcitabine; ID, investigational drug; NE, not evaluable; PHC, perihilar cholangiocarcinoma; SBS, side‐by‐side; SIS, stent‐in‐stent.

The details of the stent placement procedures are shown in Table 2. As for the endoscopists who performed the procedures, 17 (43%) and eight patients (36%) were treated by expert endoscopists, and 23 (58%) and 14 patients (64%) were treated by trainee endoscopists in the SBS and SIS groups, respectively, with no significant differences between the two groups (*p =* 0.35). For SBS deployment, the uncovered SEMSs were placed across the papilla in 37 patients (92.5%) and above the papilla in three patients (7.5%). For SIS deployment, the uncovered SEMSs were placed above the papilla in 21 patients (95.5%), and across the papilla in one patient (5.5%). Therefore, the stents were more frequently placed across the papilla in the SBS group and above the papilla in the SIS group (*p* < 0.01). Clinical success was achieved in all (100%) patients of the SBS group and all (100%) patients of the SIS group.

**TABLE 2:** Details of the biliary stenting procedure.

	SBS	SIS	*p*‐value
Endoscopists, *n* (%)			
Trainees	23 (58)	14 (64)	
Experts	17 (43)	8 (36)	0.35
Number of inserted stents, *n* (%)			
Two	37 (93)	22 (100)	0.19
Three	3 (8)	0 (0)	
Stent position, *n* (%)			
Above the papilla	3 (8)	21 (95)	<0.05
Across the papilla	37 (93)	1 (5)	
Clinical success, *n* (%)	40 (100)	22 (100)	1.00

Abbreviations: SBS, side‐by‐side; SIS, stent‐in‐stent.

Early and late AEs in the SBS and SIS groups are shown in Table 3. Early AEs occurred in 11 (28%) patients of the SBS group and two (9%) patients of the SIS group (*p =* 0.09), with a tendency toward a higher frequency of early AEs in the SBS group as compared with the SIS group. The main early AEs in the SBS group and SIS group were pancreatitis in eight patients (20%) and two patients (9%), cholecystitis in one patient (3%) and 0 patient (0%), and cholangitis in three patients (8%) and 0 patients (0%), respectively. Late AEs occurred in 10 (25%) patients of the SBS group and three (14%) patients of the SIS group, and included liver abscess, hemobilia, cholangitis, and biloma. Liver abscess occurred in five (13%) and two patients (9%), hemobilia in two (5％) and 0 patients (0%), cholangitis in three (8%) and 0 patients (0%), and biloma in 0 (0%) and one patient (5%) of the SBS and SIS groups, respectively.

**TABLE 3:** Early and late adverse events of stent deployment by the side‐by‐side and stent‐in‐stent techniques.

	SBS	SIS	*p*‐value
Early adverse events, *n* (%)	11 (28)	2 (9)	0.09
Pancreatitis, *n* (%)	8 (20)	2 (9)	
Cholecystitis, *n* (%)	1 (3)	0 (0)	
Cholangitis, *n* (%)	3 (8)	0 (0)	
Late adverse events	10 (25)	3 (14)	0.27
Liver abscess, *n* (%)	5 (13)	2 (9)	
Hemobilia, *n* (%)	2 (5)	0 (0)	
Cholangitis, *n* (%)	3 (8)	0 (0)	
Biloma, *n* (%)	0 (0)	1 (5)	

Abbreviations: SBS, side‐by‐side; SIS, stent‐in‐stent.

**FIGURE 3:**
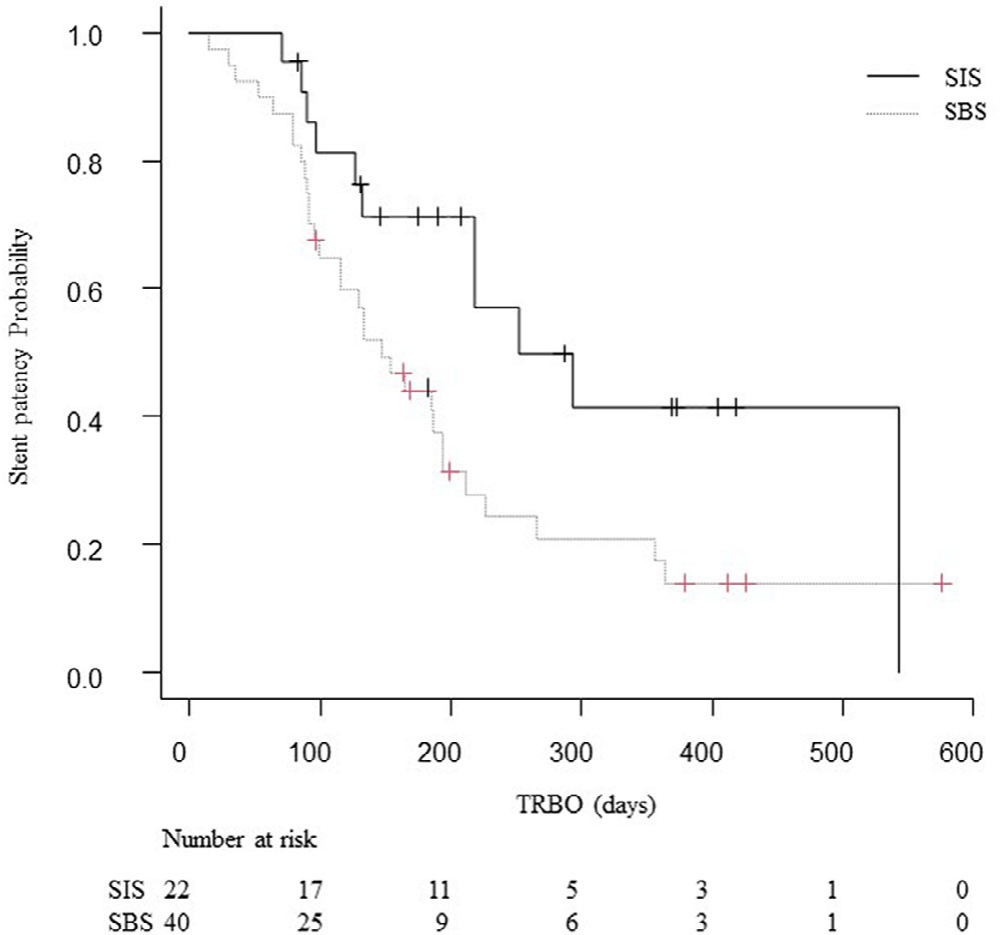
Kaplan–Meier curves for time‐to‐recurrent biliary obstruction. The median time‐to‐recurrent biliary obstruction (TRBO) was shorter in the side‐by‐side (SBS) group compared to the stent‐in‐stent (SIS) group, with medians of 147 days and 252 days, respectively (hazard ratio 2.06; 95% confidence interval 1.03–4.13; *p* = 0.029).

The clinical outcomes in the SBS and SIS groups are shown in Table 4. The RBO rates tended to be higher in the SBS group (75%) than in the SIS group (50%; *p =* 0.056). In the SBS group, there were three cases in which the stents were placed above the papilla; two of these cases developed RBO. The major causes of RBO were tumor ingrowth (38%) and sludge (33％) in the SBS group, and tumor ingrowth (32%) in the SIS group. The cumulative TRBOs are shown in Figure 3. The median TRBO was shorter in the SBS group (median: 147 days) as compared with the SIS group (median: 252 days; hazard ratio [HR] 2.06; 95% CI 1.03–4.13; *p =* 0.029). Censoring in TRBO occurred in 22 of 62 patients (35%), and RBO occurred in 40 of 62 patients (65%). There was no significant difference in the rate of conversion to percutaneous transhepatic biliary drainage after RBO (10% in the SBS group and 18% in the SIS group; *p =* 0.36), rate of conversion to endoscopic ultrasound‐guided biliary drainage after RBO (13% in the SBS group and 0% in the SIS group; *p =* 0.087), or cumulative TRBO after re‐intervention (101 days in the SBS group and 81 days in the SIS group; HR 1.64; 95% CI 0.62–4.3; *p =* 0.48) between the two groups. The total number of sessions of re‐intervention during the follow‐up was higher in the SBS group (median: 2) as compared with the SIS group (median: 1; *p =* 0.006).

**TABLE 4:** Clinical outcomes of patients undergoing stent deployment by the side‐by‐side and stent‐in‐stent techniques.

	SBS	SIS	Hazard ratio (95% CI)	*p*‐value
RBO rates, *n* (%)	30 (75)	11(50)		0.056
RBO, *n* (%)				
Sludge	13 (33)	4 (18)		0.78
Ingrowth	15 (38)	7 (32)		
Overgrowth	1 (3)	0 (0)		
Migration	1 (3)	0 (0)		
Cumulative TRBO (days)				
Median (95% CI)	147 (99–193)	252 (132–544)	2.06 (1.03–4.13)	0.029
Total number of re‐intervention sessions during follow‐up				
Median (Range)	2 (0‐12)	1 (0‐6)		0.006
Conversion to PTBD after RBO, *n* (%)	4 (10)	4 (18)		0.36
Conversion to EUS‐BD after RBO, *n* (%)	5 (13)	0 (0)		0.087
Cumulative TRBO after re‐intervention (days)				
Median (95% CI)	101 (21–872)	81 (10–615)	1.64 (0.62–4.3)	0.48

Abbreviations: CI, confidence interval; EUS‐BD, endoscopic ultrasound‐guided biliary drainage; PTBD, percutaneous transhepatic biliary drainage; RBO, recurrent biliary obstruction; SBS, side‐by‐side; SIS, stent‐in‐stent; TRBO, time‐to‐recurrent biliary obstruction.

**FIGURE 4:**
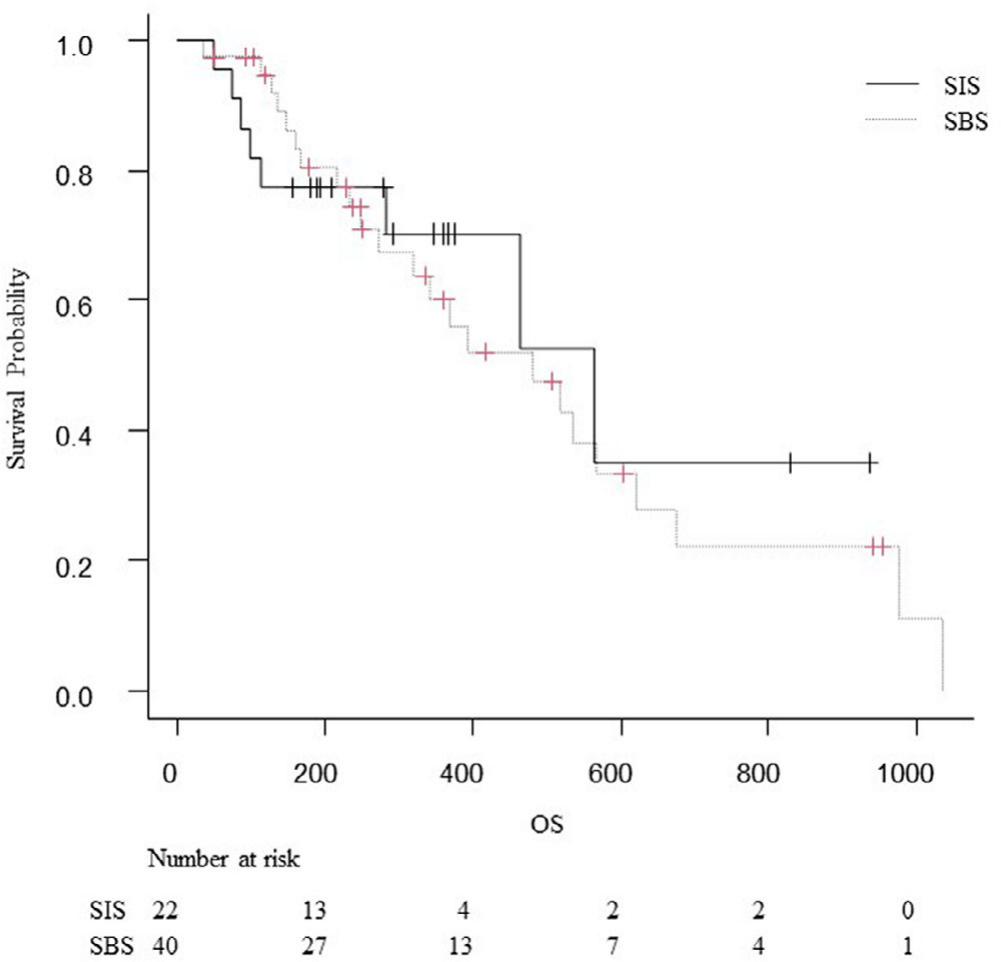
Kaplan–Meier curves for overall survival (OS). The median OS was 480 days in the side‐by‐side (SBS) group and 563 days in the stent‐in‐stent (SIS) group, with no significant difference between the two groups (hazard ratio 1.20; 95% confidence interval 0.53–2.71; *p* = 0.92).

We also assessed the TTF and rate of treatment interruption due to RBO during first‐line chemotherapy, and the results are shown in Table 5. TTF of first‐line chemotherapy was 322 days in the SBS group and 475 days in the SIS group (HR 1.09; 95% CI 0.6–1.96; *p =* 0.31). During first‐line chemotherapy, 63% of patients in the SBS group and 45% of patients in the SIS group needed interruption of chemotherapy due to RBO (*p =* 0.285). The OS are shown in Table 5 and Figure 4. The median OS was 480 days in the SBS group and 563 days in the SIS group (HR 1.20; 95% CI 0.53–2.71; *p =* 0.92), with no significant difference between the two groups.

**TABLE 5:** Clinical outcomes of patients treated by systemic chemotherapy.

	SBS	SIS	Hazard ratio (95% CI)	*p*‐value
Time‐to‐treatment failure				
Median (95% CI)	322 (126–422)	475 (161–NA)	1.088 (0.6–1.96)	0.31
Treatment interruption due to RBO, *n* (%)	25 (63)	12 (45)		0.285
Overall survival (days)				
Median (95% CI)	480 (271–620)	563 (282‐NA)	1.196 (0.53–2.71)	0.92

Abbreviations: CI, confidence interval; NA, not available; RBO, recurrent biliary obstruction; SBS, side‐by‐side; SIS, stent‐in‐stent.

We performed multivariate analysis to compare the TRBO between the groups that received stent deployment by the SBS and SIS methods, by controlling for confounding factors and using stent position, timing to chemotherapy initiation, clinical disease stage, and stenting technique as independent variables, and the results are shown in Table S1. None of the factors were identified as showing any statistically significant associations.

## DISCUSSION

We compared the duration of stent patency between unresectable BTC patients with hilar MBO who underwent stent deployment by the SBS and SIS techniques during systemic chemotherapy; the results revealed that the duration of stent patency was significantly shorter in the SBS group than in the SIS group. Previously, SEMSs in the SBS group were frequently placed across the papilla due to kinking risk and re‐intervention difficulty,^9^ leading to more duodenal reflux and sludge retention. On the other hand, the SEMS were more often implanted above the papilla in the SIS group; as the SEMSs were overlapping in the common bile duct and re‐intervention was not difficult even when the stents were placed above the papilla. Less exposure of the stent to the duodenal lumen likely reduces the risk of obstruction from food residue and sludge retention, contributing to a longer duration of stent patency. This might contribute to the longer‐term stent patency. Furthermore, in the SIS group, the stents were overlapping in the common bile duct, therefore the stents could be placed in a natural manner with no overexpansion, which could have contributed to the longer stent patency and lower need for re‐intervention compared to the SBS group. Recent meta‐analyses reported a shorter duration of patency and higher re‐intervention rates for stent deployment by the SBS method as compared with the SIS method, although the superiority of either technique remains under debate.^11^ The rate of early AEs rate tended to be higher in the SBS group, although there was no significant difference in the rate of late AEs between the two groups. SBS stent placement involves parallel bile duct stenting, which leads to early complications due to overexpansion of the bile duct. This finding of ours was also consistent with previous reports.^4^, ^11^

However, this study included a mix of patients with a variety of cancer types who had and had not received chemotherapy, which made it difficult to determine the exact duration of stent patency in chemotherapy‐eligible biliary tract cancer patients; it was also not possible to determine the effect of the stent deployment technique on the possibility of continuation or need for discontinuation of chemotherapy.

We examined the effect of first‐line chemotherapy according to the stenting method used. Treatment interruption due to RBO was more frequent and the TTF was slightly shorter in the SBS group than in the SIS group. These findings suggest that systemic chemotherapy could be continued for longer without interruption by RBO in the SIS group. Although there are no studies that examined the effect of stenting format on chemotherapy in biliary tract cancer during chemotherapy, there are some studies that compared the duration of patency of plastic and covered metal stents in pancreatic cancer during chemotherapy and reported that the metal stents had less occlusion and longer patency.^14^, ^15^ The selection of appropriate stenting is considered important for the long‐term continuation of chemotherapy.

RBO occurring in the short term might necessitate interruption or discontinuation of chemotherapy. Longer patency durations of stents could also improve the patient's quality of life by reducing the number of re‐interventional procedures and hospitalizations required due to RBO. Until now, there have been few studies on the stent patency duration in unresectable BTC patients receiving systemic chemotherapy, and prior studies on the subject were limited by the varied cancer types and chemotherapy status of the patients.^8^, ^9^, ^10^, ^11^ To address these issues, this study was limited to patients with biliary tract cancer before or immediately after the introduction of first‐line chemotherapy. The results of this study revealed that in patients undergoing systemic chemotherapy for unresectable BTC, stent insertion by the SIS technique yielded longer stent patency as compared with stent insertion by the SBS technique, consequently allowing for long‐term continuation of systemic chemotherapy without interruption or discontinuation due to the development of RBO.

Our study had limitations. First, it was a single‐center retrospective study. Because this was not a randomized controlled trial and was conducted on a rather small number of cases, the possibility of unintentional selection bias of patients cannot be fully excluded. Secondly, this study included patients who underwent multi‐stenting before the first or second cycle of first‐line chemotherapy to ensure the number of cases, it would be preferable to focus on patients in which the stent was inserted before the start of chemotherapy to examine TRBO more accurately. Thirdly, the chemotherapy regimens were varied, which may prevent an accurate comparison of the results. In addition, treatment with GC + durvalumab and GC + pembrolizumab, which are standard regimens for unresectable BTC at present, had not yet been approved during this study period in Japan. Therefore, these treatments were not included in our analysis. Nevertheless, our findings can be examined in future randomized controlled trials with uniformity regarding the type of chemotherapy in patients who had a stent inserted before chemotherapy, which will provide more reproducible results on the duration of stent patency during chemotherapy. Fourthly, we often placed the SEMS across the papilla in the SBS group; the location of the distal end of the stent is undoubtedly an important factor with respect to the TRBO, and multivariate analysis performed with the stent position included as a variable did not show any advantage for SIS. However, the deployment method could vary among institutions, potentially limiting its generalizability.

In conclusion, in patients with unresectable BTC receiving systemic chemotherapy, the stent patency duration was significantly shorter in patients who underwent stent insertion by the SBS technique rather than by the SIS technique. Furthermore, stent placement by the SIS technique was associated with a reduced need for re‐intervention and chemotherapy interruptions and was preferred over stent placement by the SBS technique.

## CONFLICT OF INTEREST STATEMENT

Masafumi Ikeda has received research funding (institution) from AstraZeneca, Bayer, Bristol Myers Squibb, Boehringer Ingelheim, Chugai, Chiome Bioscience, Delta‐Fly Pharma, Eisai, Eli Lilly Japan, Invitae, MSD, J‐Pharma, Merck biopharma, Merus N.V., Novartis, Nihon Servier, Ono, Pfizer, and Syneos Health, consulted for AstraZeneca, Chugai, MSD, Nihon Servier, and Novartis, and received speaker honoraria from AbbVie, AstraZeneca, Chugai, Eisai, Eli Lilly Japan, Fujifilm Toyama Chemical, Guardant Health Japan, Incyte Biosciences Japan, MSD, Nihon Servier, Novartis, Nippon Kayaku, Ono, Taisho Pharmaceutical, Teijin, Takeda, Taiho, and Yakult. Shuichi Mitsunaga has received research funding from Chugai, Astellas, Toray, Ajinomoto, and Pfizer, and received speaker honoraria from Ono, Toray, and Otsuka. HI has received research funding from Ono, and Novartis, consulted for Nihon Servier, and Kaneka Medix, and received speaker honoraria from Yakult, AstraZeneca, Nihon Servier, Kaneka Medix, Boston Scientific, and SB‐Kawasumi Laboratories. The other authors declare no conflict of interest.

## ETHICS STATEMENT

The study protocol was approved by the Institutional Review Board of the National Cancer Center.

## PATIENT CONSENT STATEMENT

N/A.

## CLINICAL TRIAL REGISTRATION

N/A.

## Supporting information




**TABLE S1** Multivariate analysis to identify factors influencing the TRBO.
