# Supplementary material for: Side‐by‐side versus stent‐in‐stent technique for stent deployment during systemic chemotherapy in biliary tract cancer patients with malignant hilar biliary obstruction
Source: DEN Open. 2025 Feb 7;5(1):e70075. doi: 10.1002/deo2.70075 (PMC11803297; doi:10.1002/deo2.70075)
Supplement: Supplementary file 1 — TABLE S1 Multivariate analysis to identify factors influencing the TRBO. [file DEO2-5-e70075-s001.docx]

**Supplemental Table 1. Multivariate analysis to identify factors influencing the TRBO**

| **Factors** | **Multivariate analysis** | |
| --- | --- | --- |
|  | **Hazard Ratio**  **(95% CI)** | **P-value** |
| Across the papilla | 1.00  (0.30-3.34) | 1.00 |
| Stenting prior to the initiation of chemotherapy | 0.70  (0.27-1.82) | 0.46 |
| Stage, metastatic | 1.53  (0.76-3.06) | 0.23 |
| SBS technique | 2.29  (0.64-8.16) | 0.20 |

TRBO, time-to-recurrent biliary obstruction; CI, confidence interval; SBS, side-by-side
